# Coupling between Intrinsic Prefrontal HbO2 and Central EEG Beta Power Oscillations in the Resting Brain

**DOI:** 10.1371/journal.pone.0043640

**Published:** 2012-08-24

**Authors:** Gert Pfurtscheller, Ian Daly, Günther Bauernfeind, Gernot R. Müller-Putz

**Affiliations:** Institute for Knowledge Discovery, Laboratory of Brain-Computer Interfaces, Graz University of Technology, Graz, Austria; Hangzhou Normal University, China

## Abstract

There is increasing interest in the intrinsic activity in the resting brain, especially that of ultraslow and slow oscillations. Using near-infrared spectroscopy (NIRS), electroencephalography (EEG), blood pressure (BP), respiration and heart rate recordings during 5 minutes of rest, combined with cross spectral and sliding cross correlation calculations, we identified a short-lasting coupling (duration 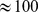 s) between prefrontal oxyhemoglobin (HbO2) in the frequency band between 0.07 and 0.13 Hz and central EEG alpha and/or beta power oscillations in 8 of the 9 subjects investigated. The HbO2 peaks preceded the EEG band power peaks by 3.7 s in 6 subjects, with moderate or no coupling between BP and HbO2 oscillations. HbO2 and EEG band power oscillations were approximately in phase with BP oscillations in the 2 subjects with an extremely high coupling (squared coherence 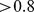) between BP and HbO2 oscillation. No coupling was identified in one subject. These results indicate that slow precentral (de)oxyhemoglobin concentration oscillations during awake rest can be temporarily coupled with EEG fluctuations in sensorimotor areas and modulate the excitability level in the brains’ motor areas, respectively. Therefore, this provides support for the idea that resting state networks fluctuate with frequencies of between 0.01 and 0.1 Hz (Mantini et.al. PNAS 2007).

## Introduction

One of the major tasks of the brain is to organise, control and execute motor behaviour. For this, two cortical regions are of special importance, the prefrontal cortex and the primary motor cortex. The former plays a role in the conscious experience of intending to act, the latter in the execution of the motor act [1].

The prefrontal cortex is on the summit of the cortical motor hierarchy and is widely connected with many other brain structures, cortical and sub-cortical. In the case of self-paced movement, activation spreads from the prefrontal cortex via the premotor areas to the primary motor cortex. In the course of movement preparation the Bereitschaftspotential is generated [2] and the sensorimotor rhythms are desynchronised (premovement event-related desynchronisation, (ERD) [3]), additionally the heart rate is decreased ([4],[5],[6]). All these phenomena are measurable a few seconds prior to movement onset and act as a good example of the mutual interaction between the brain and the heart, as first postulated by Claude Bernard about 150 years ago ([7] pp. 71–72, originally published in 1872). Functional magnetic resonance imaging (fMRI) studies have shown that the decision to perform either a self-paced left or right hand movement can be encoded in the prefrontal cortex up to 10 s before it enters awareness [8]. This relatively long time span and the beginning of the heart rate (HR) deceleration a few seconds prior to movement needs special attention.

An interesting property of the brain is the slow fluctuations of fMRI blood oxygenation level-dependent (BOLD), near-infrared spectroscopy (NIRS) and electroencephalogram (EEG) signals. Although slow and infraslow fluctuations are a prominent feature in EEG, BOLD and NIRS signals, we still do not know where they originate. There are different possibilities, such as, for example, the firing of neurons in the reticular formation of the brain stem, which waxes and wanes with a period of 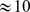 s during wakefulness and sleep [9]. Alternatively, neurons may slowly modulate their activity level because of intrinsic excitability changes [10]. Additionally, slow systemic fluctuations due to the dynamics of cerebral autoregulation may also play a role [11]. Fluctuations around 0.1 Hz in the resting brain found in the EEG ([12],[13]) and in the oxyhemoglobin (HbO2) and deoxyhemoglobin (Hb) concentrations ([14],[15],[16]) are of special interest.

Interestingly, intrinsic activity, as measured with fMRI-BOLD and EEG in the resting brain, is organised in multiple highly specific functional anatomical networks that fluctuate at frequencies between 0.01 and 0.1 Hz [17]. Information about the coupling of cortical networks can be obtained not only from fMRI studies but also by the study of temporal correlation between 2 hemodynamic signals (e.g. HbO2 and Hb) or between hemodynamic and EEG band power signals in low frequency ranges. For example, the phase-shift between HbO2 and Hb oscillations around 0.1 Hz in the prefrontal cortex depends on the mental workload [15] and the fronto-posterior connectivity in the resting state is significantly higher for HbO2 compared to Hb oscillations around 0.1 Hz [16]. Of additional interest is the observation that slow prefrontal HbO2/Hb oscillations can be phase coupled with the slow BP oscillations with a similar phase shift during rest and movement tasks [18].

A close interaction exists between the prefrontal cortex and the primary motor cortex during movement tasks. It is, therefore, of interest to ask whether some functional coupling between both cortical areas also exists in the resting brain. Thus, we performed a combined NIRS and EEG study to explore the correlations between slow prefrontal HbO2/Hb oscillations and central alpha and beta power fluctuations during rest. From [18] and another recently published paper [16] there is evidence that 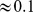 Hz Hb and HbO2 concentration oscillations during rest are rare and found only in a small group of subjects.

We recently reported the novel finding of coupling between prefrontal HbO2 fluctuations and central EEG band power changes during rest [19]. This study was preliminary with the following limitations: (i) EEG data were band pass filtered in subject-specific most reactive alpha and beta frequency bands determined in the movement session, (ii) segments with relatively clear HbO2 waves were selected by visual inspection of the raw data, (iii) consecutive positive HbO2 peaks in the selected segment were marked and used as triggers for averaging over aligned segments of HbO2, Hb and EEG band power, and (iv) determination of the delays between the positive peaks of the averaged HbO2 and EEG band power changes. To obtain support for this coupling we used a different methodology in this paper : (i) the use of the same frequency bands in each subject, (ii) the use of sliding cross correlation analysis to investigate the coupling between prefrontal HbO2 fluctuations and EEG band power changes, (iii) determination of the delay between HbO2 and EEG oscillations from the cross correlation function and (iv) application of appropriate statistical tests to confirm the presence of transient periods of coupling between HbO2 and EEG. A further goal of this study was to relate the EEG band power fluctuations during rest to concomitant slow oscillations in HbO2, BP and heart rate.

## Methods

The group of subjects, the recording condition and the coupling between slow (de)oxyhemoglobin and blood pressure oscillations have been described in detail in a previous paper [18]. The phase coupling was estimated in 19 subjects via cross-spectral analysis between BP, HR, and NIRS signals during 5 minutes of rest with special emphasis on the coupling between HbO2 and Hb concentration changes. A reliable estimated phase can be expected when the coherence is high (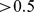; see [20]). In the case of HbO2 and Hb oscillations only 9 subjects displayed such a reliable estimated phase coupling (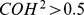) with a mean (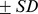) of 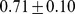 (the other 10 subjects displayed a mean (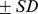) of 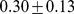). These nine subjects were used for further analysis.

### Subjects and Experimental Paradigm

The investigation was carried out in 9 naive, healthy subjects (2 male, 7 female) aged 20 to 31 years (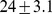, mean 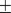 SD). All subjects displayed phase coupled slow oxy- and deoxyhemoglobin oscillations during rest (for details see [18]). The subjects all voluntarily participated in a study with an initial data recording during 5 minutes of rest without any instruction. All subjects were right-handed (Edinburgh- Handedness-Inventory (EHI)), had normal or corrected to normal vision and were seated in a comfortable armchair for the experiments. The experiments were in compliance with the World Medical Association Declaration of Helsinki. The protocol was approved by the Ethics committee of the Medical University of Graz, and the subjects gave informed written consent before participating.

### Data Recording and Pre-processing

We recorded ECG, blood pressure, (de)oxyhemoglobin, respiration and EEG. All signals were sampled at a frequency of 500 Hz. ECG was recorded bipolarly at electrodes placed on the thorax (filter setup: 0.5–100 Hz). Blood pressure was recorded continuously via a non-invasive monitoring system (CNAP Monitor 500, CNSystems, Austria) from the proximal limb of the index or middle finger. The respiration patterns were obtained by using a respiratory sensor (Respiratory Effort Sensor, Pro-Tech Services Inc., filter setup: 0.1–100 Hz).

Three EEG signals were recorded (0.5–100 Hz) bipolarly over the three primary motor areas (left hand, foot and right hand representation areas) of the sensorimotor cortex (electrodes were placed 2.5 cm anterior and posterior to C3, Cz and C4) using a biosignal amplifier (g.BSamp, g.tec medical engineering GmbH, Austria). (De)oxyhemoglobin concentration changes were recorded with a custom made one-channel, continuous wave method-based NIRS system (for details see [21]). The sources and the detector were placed over the left lateral prefrontal cortex (Brodmann area 10, this area is responsible for the temporary organisation of movement including the prefrontal function of readiness to act [22]) 1.5 cm to the left and right of position FP1 according to the international 10/20 system for EEG recording. A fifth-order Butterworth filter with a cut-off frequency of 0.9 Hz was used to remove variability due to the cardiac cycle.

### ECG and BP Processing

**Figure 1:**
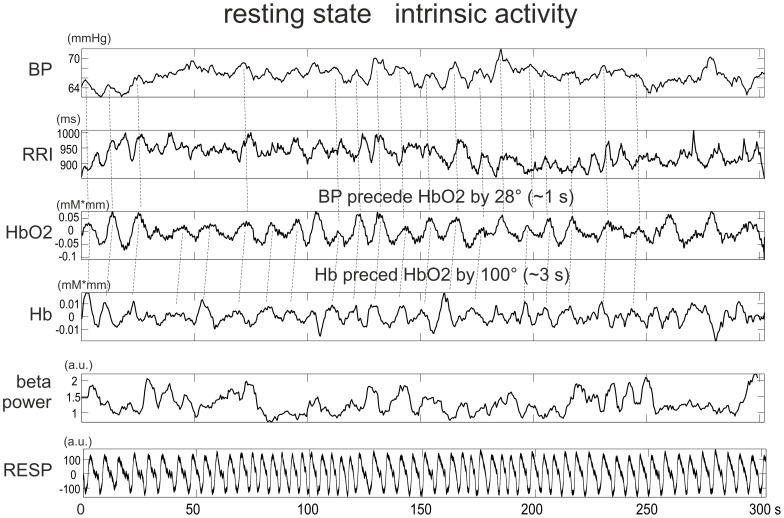
Data from a representative subject (S8). From top to bottom: time series of BPdia (mmHg), RRI (ms), HbO2 (mM*mm), Hb (mM*mm), EEG band power (C4, 15–25 Hz), and respiration (a.u.) during 300 s of rest. Relationships between individual peaks are marked by stippled lines.

After detection of the beat-to-beat intervals (RRI) in the ECG signal (based on an algorithm using a filter bank to decompose the ECG signal into various subbands), the intervals were linearly interpolated, resampled at 2 Hz, and displayed as RRI time series. From the arterial blood pressure recording, the systolic (BPsys) and diastolic (BPdia) pulse pressure amplitudes were extracted, linearly interpolated, resampled at 2 Hz, and displayed as BPsys and BPdia time series. A transfer function model [5] was used to remove respiratory-related variability from the instantaneous RRI- and BP-time series. Examples of such time series, together with NIRS and EEG power signals, and respiration are shown in Fig. 1.

### Estimation of Coherence and Phase Spectra

Cross-spectra were calculated for the BP and RRI time series and HbO2 (Hb) signals resampled at 2 Hz. The spectral values of 1024 samples were smoothed using a 31-point triangular window (for details see [18], [20], and [23]). After an automatic search for the largest peak in the cross-spectrum in the range 0.07–0.13 Hz, the corresponding squared coherence (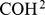) and phase-shift (PHA) values were determined. The frequency of the peak was termed dominant frequency (DF) and named DF1 for the dominant frequency of HbO2 vs. Hb cross spectra, DF2 for HbO2 vs. BP spectra and DF3 for BP vs. RRI spectra.

### EEG Processing

The EEG signals were band pass filtered in the frequency bands 9–14 Hz and 15–25 Hz, the samples squared and then low pass filtered with a fourth-order Butterworth filter (cut-off frequency 0.13 Hz). The resulting band power time courses were log transformed and resampled at 2 Hz. The signals were segmented via a sliding window approach with a window length of 100 s and step size of 50 s. The cross correlation between EEG band power and HbO2 signals was calculated for every window and the largest peak was identified in the cross correlation plot 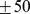 s, this was used to calculate the delay between the two signals. The signals were offset against one another by this delay and the correlation calculated.

### Statistical Analysis

Relationships between HbO2/Hb changes within the prefrontal cortex and EEG beta band activity within the primary motor areas were measured via the correlation coefficient. Because the nature of the correlation appears to change dynamically over time the correlation was calculated within a sliding window of length 100 s and step size 50 s. These values were chosen to provide a balance between the need for a short window length to give accurate measures of the dynamics of the changes in correlation between the signals and a sufficiently long window length to allow accurate estimation of the correlation coefficients.

Statistical significance of the correlation between the HbO2 and EEG signals was estimated via a bootstrapping approach. The signals within each 100 s window are assumed to be locally stationary and bootstrap replications of the signals within each window were generated directly from the signals within each window. To test the assumption of local linearity within the windows and global non-linearity over the whole signals non-linearity tests based upon estimates of the third order moments of the time series, as proposed in [24], were applied to each window and the whole signals.

The traditional approach towards bootstrapping is to generate bootstrap replications of each dataset under a suitable null hypothesis. In the case of correlation, a suitable null is that the relationship between the signals is random. To generate bootstraps under this null, sample by sample shuffling may be used. However, sample-wise shuffling assumes consecutive samples to be independent. This is not the case in EEG or NIRS signals and so a different approach is required. The recommended approach is therefore to use a moving block bootstrap, in which the signal is segmented into consecutive blocks which are then shuffled [25]. Doing so maintains, on average over multiple bootstraps, the sample to sample dependencies, while breaking the relationships between the signal pair.

However, this approach raises the question of how one chooses the block length. A proposed solution is to draw the block length from a geometric distribution, referred to as the stationary bootstrap [26]. This has the advantage of generating bootstrap replication signals which are automatically centered on the sample mean and have been demonstrated to have good consistency and asymptotic accuracy.

Therefore, the stationary bootstrap approach is used in this work. In total 4,000 bootstraps are generated for each window position from the EEG and NIRS signals within the windows. The significance of the correlation between the original signals is then estimated against the distribution of correlation values estimated from the bootstrap replications. Finally, Bonferroni correction for multiple comparisons was applied as an additional check for the validity of the statistical significance.

## Results

### Coupling between Prefrontal Hemodynamic and Central EEG Power Fluctuations

**Figure 2:**
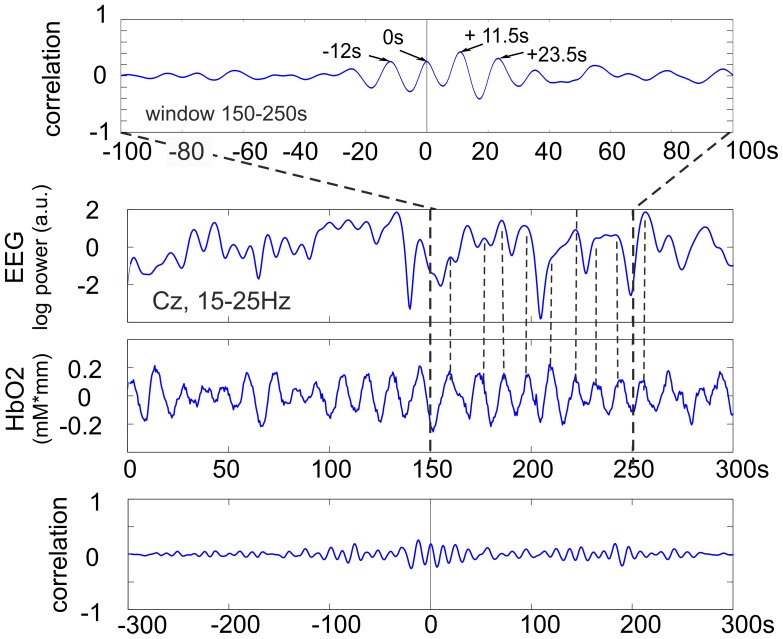
From top to bottom. cross correlation function (CCF) for the 150–250 s window, time courses of EEG log band power (Cz, 15–25 Hz) and HbO2 during 300 s of rest and CCF calculated from the total 300 s (5 minute) epoch. Subject S2. Note, the significant correlation (r = 0.49, , p<0.01) in the 100 s window and the non-significant correlation (r = 0.2) for the total 300 s.

**Figure 3:**
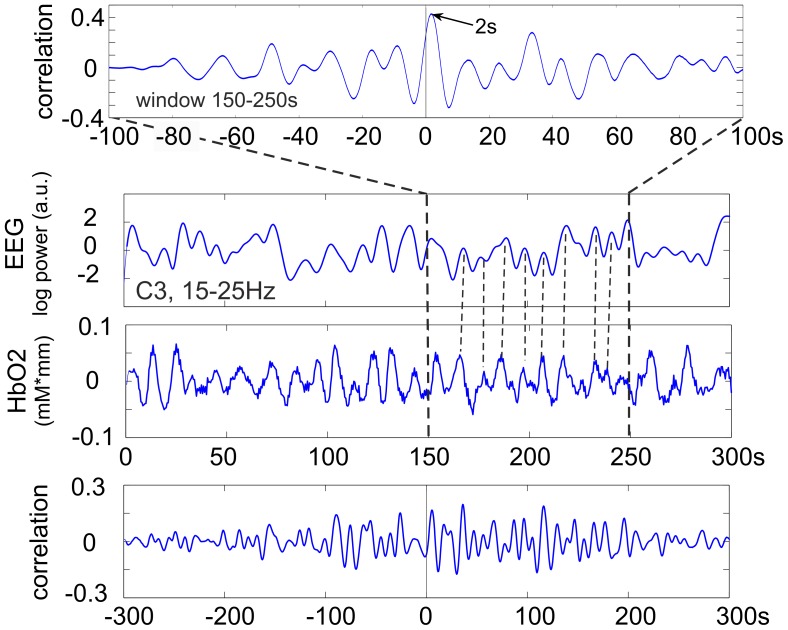
From top to bottom. cross correlation function (CCF) for the 150–250 s window, time courses of EEG log band power (C3, 15–25 Hz) and HbO2 during 300 s of rest and CCF calculated from the total 300 s epoch. Subject S8.

The assumption of local linearity and global non-linearity is borne out. 2% of 100 s segments are seen to be non-linear while 87% of the whole signals are seen to be non-linear. The coupling results of all subjects (maximal correlation, lag, significance) are summarised in Table 1 and examples of cross correlation functions from 2 representative subjects are shown in Figs. 2 and 3. The sliding cross correlation analyses revealed, in 8 subjects, a significant (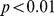) correlation (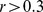) between 100 s segments of HbO2 and EEG-band power with a preference to channels C3 and Cz and a slight preference for the 15–25 Hz band. Remarkably, with the exception of one measure, the correlation peak delays were all positive, meaning that HbO2 concentration changes preceded the EEG band power changes. On average this delay (with the exception of 2 subjects indicated in Table 1) was 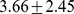 s. In the majority of subjects only one 100 s segment revealed a significant coupling.

**Table 1:** Correlations between EEG and HbO2 for all subjects.

			C3		Cz		C4		
	Frequencyband	Window*p*<0.01	delay	corr.	delay	corr.	delay	corr.	delay
	Hz	s	s		s		s		s
S1	9–14	–	–	–	–	–	–	–	
	15–25	0–100	3.5	0.33	–	–	–	–	3.5
S2	9–14	–	–	–	–	–	–	–	
	15–25	150–250	9.5	0.36	11.5	0.49	–	–	11.5/0.0
S3	9–14	100–200	−0.5	0.31	0.5	0.36	0.5	0.36	0.5
	15–25	–	–	–	–	–	–	–	
S4	9–14	200–300	8.0	0.33	–	–	–	–	
	15–25	200–300	8.5	0.38	–	–	–	–	8.5
S5	9–14	50–150	–	–	–	–	11.0	0.38	
	15–25	50–150	–	–	–	–	10.5	0.36	10.5/2.0
S6	9–14	150–250	–	–	2.0	0.32	–	–	2.0
	15–25	–	–	–	–	–	–	–	
S7	9–14	200–300	–	–	11.5	0.31	–	–	
	15–25	0–100	5.5	0.33	–	–	–	–	5.5
S8	9–14	0–100	–	–	4.5	0.31	–	–	
	15–25	150–250	2.0	0.41	–	–	–	–	2
S9	9–14	–	–	–	–	–	–	–	
	15–25	–	–	–	–	–	–	–	
Mean									3.66
SD									2.45

For each subject 100 s windows, which exhibit significant correlation (r>0.3), and their corresponding delays, are listed in the 9–14 and 15–25 Hz frequency bands. In the last column the delays with the largest correlation are indicated. For subject’s S2 and S5 the second largest correlations are also marked. Mean delay (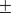 SD) is calculated without subject’s S2 and S5. Subjects with a high coupling (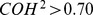 between HbO2 and BP are highlighted in grey.

Subjects S2 and S5 need special attention, both exhibit oscillating cross correlation functions (CCFs) (for an example see Fig. 2). The former with a correlation peak delay of 11.5 s and the latter with a delay of 10.5 s (see Table 1). From the CCF in Fig. 2 (subject S2) it can be interpreted that both the EEG beta power and HbO2 oscillations are in-phase in the window 150–250 s and that the HbO2 precedes the EEG oscillations by one period, namely 11.5 s. In subject S5 the CCF also displayed an oscillating behaviour and showed peaks at 2 s (non-significant) and 10.5 s (significant; 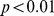, see Table 1). In this case the HbO2 preceded the EEG oscillations either by 2 s or more likely by 10.5 s (the difference of 10.5 s minus 2 s = 8.5 s corresponds to the period of the HbO2 oscillations).

In subject S8, another representative subject, the CCF displayed a correlation peak delay of 2 s (Fig. 3) meaning that the HbO2 preceded the EEG oscillations by 2 s in the window 150–250 s.

### Coupling between Slow BP, RRI, HbO2 and Hb Oscillations

For the interpretation of the coupling between slow HbO2 and EEG power oscillations, the average phase coupling between the HbO2 and Hb, HbO2 and BP time series, and BP and RRI time series, are of interest. The dominant frequencies were determined for HbO2/Hb oscillations (DF1), HbO2/BP oscillations (DF2) and BP/RRI oscillations (DF3). The different dominant frequencies and phase couplings are summarised in Table 2. All subjects except one (S7) displayed similar DFs for HbO2/Hb and BP/RRI oscillations (differences between DF1, DF2 and DF3 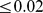 Hz). In subject S7 no coupling (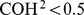) was found between prefrontal HbO2 and BP oscillations, due to the different dominant frequencies of BP and HbO2 oscillations.

**Table 2:** For each subject, couplings are listed between, HbO2 and Hb, HbO2 and BP, and BP and RRI.

	Coupling HbO2 vs Hb					Coupling HbO2 vs BP				Coupling BP vs RRI			
	DF1	Period DFp	phase shift		COH^2^	DF2	phase shift		COH^2^	DF3	phase shift		COH^2^
	Hz	s	grad	s		Hz	grad	s		Hz	grad	s	
S1	0.09	10.9	−201	−6.1	0.63	0.10	−166	−4.6	0.59	0.09	−62	−1.9	0.72
S2	0.08	12.2	−20	−0.7	0.87	0.08	−46	−1.6	0.95	0.08	−86	−3.0	0.91
S3	0.08	11.9	−80	−2.6	0.69	0.09	−99	−3.1	0.55	0.08	−52	−1.8	0.61
S4	0.11	9.2	−67	−1.7	0.55	0.11	−58	−1.5	0.65	0.11	−83	−2.1	0.77
S5	0.11	8.8	−14	−0.3	0.83	0.12	−14	−0.3	0.92	0.12	−53	−1.2	0.84
S6	0.08	11.9	−18	−0.6	0.70	0.07	−2	−0.1	0.54	0.09	−69	−2.1	0.48
S7	0.08	12.2	0	−0.0	0.66	–	–	–	–	0.13	−109	−2.3	0.74
S8	0.10	10.2	−101	−2.9	0.64	0.09	−28	−0.9	0.62	0.09	−91	−2.8	0.79
S9	0.12	8.3	−7	−0.2	0.76	0.12	−69	−1.6	0.70	0.12	−89	−2.1	0.81
mean	0.10	10.62	−56.44	−1.67	0.70	0.10	−60.25	−1.70	0.69	0.10	−77.11	−2.15	0.74
SD	0.01	1.55	64.99	1.96	0.10	0.02	52.84	1.49	0.16	0.02	19.27	0.52	0.13

Dominant frequencies (DFs), periods, phase shifts, and squared coherence values (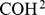) are listed in each case. Subjects with a high coupling (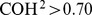) are highlighted in grey.

Inspection of Table 2 reveals that 2 groups of subjects can be identified, subjects with a high coupling between slow autonomic BP and slow central HbO2 oscillations (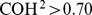; S2, S5, S9) or no coupling (S7), and subjects with a moderate coupling (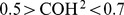; S1, S3, S4, S6, S8). In the group with a high coupling the phase shifts between HbO2 and Hb oscillations were small (<−20° and <0.7 s, respectively). In the group with a moderate coupling the phase shifts between HbO2 and Hb oscillations were moderate to large (>60°). Remarkably, in 4 of these subjects (S1, S4, S6, and S8) the Hb preceded not only the HbO2 but also the BP oscillations.

## Discussion

### Reliability of the Cross Correlation

Although it’s well known that non-linear components play a role in the generation of brain (e.g. [13] and [27]) and cardiovascular rhythms (e.g. [28] and [29]) we used, in a first step, linear methods and thereby verified the presence of coupling between slow fluctuating hemodynamic and EEG signals. The next step in our research will be to plan a multichannel NIRS and EEG recording with the use of non-linear analysis methods. In particular, the observation that Hb frequently displays an oscillatory behaviour around half the frequency of the HbO2 oscillations (e.g. 0.05 Hz vs. 0.1 Hz) is an indicator of non-linearity.

The EEG and physiological processes are known to be non-linear processes with high degrees of inter-subject variability [30]. It is, therefore, unsurprising that the relationship between physiological processes should be non-stationary, with short lived periods of significant correlations occurring at different times between the EEG and HbO2 signals. Correlation is a linear method, that is, it attempts to measure the degree of a linear, stationary relationship between a pair of signals. Therefore, to measure a non-stationary relationship with the correlation function it is necessary to apply a sliding window approach, as is done in this work, with the assumption that the relationship is transiently stationary (stationary within the window). The choice of the window length and step size are based upon visual observations of apparent relationships between the signals (see [18] for further details). Thus, this work may be seen as a statistical verification of visually apparent relationships between the EEG and HbO2 processes. The sliding window is necessary to account for the non-stationarity of the signals, but it does not solve the question of non-linearity. To solve the latter other methods have to be used, such as Mutual Information, bicoherence or non-linear association measures.

It may be of interest, in future work, to make a more detailed analysis of the non-linear dynamical relationships between the physiological signals. For example, the phase locking value coupled with hidden markov models (HMMs) could be used to do this, as is the case in e.g. [31].

### Correlation between Prefrontal Hemodynamic and Central EEG Band Power Changes

The main finding of the present study was the temporary coupling of slow prefrontal HbO2 oscillations with similar fluctuations of EEG alpha and/or beta power in sensorimotor areas in the resting brain. Temporary means that, in most subjects, slow HbO2 oscillations revealed a significant correlation (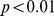) with EEG band power oscillations for only approximately 100 s intervals. In some subjects a significant correlation was also found for longer periods. Some explanation is needed for the correlation findings in the two representative subjects S2 and S8. In both cases the correlation was significant; in S2 the correlation function was in-phase with the HbO2 and EEG changes (Fig. 2) but in S8 it was delayed by 2 s (Fig. 3).

The coupling, for only short time windows, between prefrontal hemodynamic and central electrical oscillations is not unexpected. There is a large body of literature ([9],[13],[17],[32],[33],[34]) reporting on EEG fluctuations ranging from ultraslow (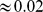 Hz) to slow (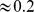 Hz) and on nonstationarities of correlated hemodynamic (fMRI BOLD signal), heart rate, and EEG alpha power fluctuations of the brains resting state (see e.g. [35]). This means the intrinsic brain activity is never stationary and is also closely linked to slow changes of the hearts beat-to-beat intervals (RRI). The latter are modulated not only by pathways originating in the prefrontal cortex [36] but also by the slow BP waves of the baroreflex loop [37] whereby these waves precede the RRI waves by 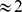 s [18], [20], see also (Table 2).

### Relationships between HbO2-EEG Coupling and Slow Oscillations of HbO2, Hb, BP and RRI

The 2 subjects with an extremely high coupling (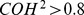) between HbO2 and BP (S2, S5) and between HbO2 and Hb oscillations also displayed high coupling between BP and RRI oscillations (see Table 2). In these two subjects the central EEG band power displayed, for 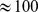 s, a significant (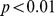) coupling with the prefrontal HbO2 oscillations in the form of cyclic amplitude changes of EEG power with a frequency of 0.08 Hz and 0.11 Hz, respectively. From this it can be interpreted that weak periodic fluctuations of the central EEG power were temporarily related to autonomic blood pressure oscillations with a lag of 13.1 s (11.5+1.6 s) in subject S2 and a lag of 2.3 s (2+0.3 s) and 10.8 s (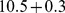 s), respectively in subject S5.

In other words, in the case of an extremely high phase coupling between HbO2 and BP oscillations and a phase shift close to zero between Hb and HbO2 oscillations, cerebral blood volume (CBV) oscillations of large magnitudes are dominant [38]. Therefore, it can be assumed that the blood pressure (Mayer waves) is the driving force for common fluctuations of the cerebral HbO2, Hb, and EEG band powers.

In the case of a moderate phase coupling between HbO2 and BP and a moderate to large phase shift between Hb and HbO2 oscillations we can assume the dominance of cerebral blood flow (CBF) and oxygen consumption fluctuations, respectively. This may be characteristic of a neurovascular coupling [38]. The existence of some coupling can be supposed in all of the subjects with a significant correlation between prefrontal oxyhemoglobin and central EEG power changes. Such a coupling is not only found in the awake state in adults but also in preterm infants during quiet sleep [14]. In pre-term and full-term infants the occurrence of EEG bursts can display a periodic pattern with inter-burst-intervals between 10–15 s, dependent on the conceptual age ([13], [32]) and is associated with delayed changes of Hb and HbO2 concentrations.

At this time it’s not completely clear how the frequency of the slow blood pressure waves (Mayer waves) can be explained. Two theories have been proposed: the central pacemaker theory ([39],[40],[41]) and the baroreflex loop theory ([20], [37]) For a detailed discussion see [42]. Support for a central oscillator (pacemaker theory) is given by the slight lead in slow Hb oscillations before BP oscillations, as found in 4 subjects. Clear support of the baroreceptor theory is given by the dominance of CBV oscillations, found in 2 subjects.

Some comment is needed on the fact that no EMG was recorded. From the EEG/MEG work of Claus et al. [43] we know that the frontal muscles show a peak in the 20–30 Hz band and temporal muscles in the 40 Hz band and above. Due to the location of our electrode positions over midcentral and temporal areas and the analysis in the 15–25 Hz band spurious results are unlikely. Also, the similar results reported in the alpha and beta bands rules out a significant EMG contribution in EEG signals.

The large variability in the HbO2-EEG couplings, as documented in Table 1, is not completely unexpected, when the non-linearity of the involved cerebral and cardiovascular systems ([27], [28], [29]) and the complexity of interactions between slow EEG [12], cerebral (de)oxyhemoglobin [16], and cardiovascular (Mayer waves) fluctuations in the resting state are taken into consideration. The dominant frequency of HbO2 oscillations varied not only from subject to subject (0.08 to 0.12 Hz, see Table 2) but also within subjects and the coupling between BP and HbO2 varied between close to 1 (S2, S5) and close to zero (S7). Indeed, it could be argued that the large variability in the results could be reduced by controlling for specific psycho-physiological factors in the subjects. However, the exploratory nature of this research entailed the uncontrolled recruitment of subjects for this study.

Additionally, the phase delay between HbO2 and Hb oscillations was large (e.g. ∼180° in S1), moderate (S3, S4, S8) or close to zero (S2, S5, S6, S7, S9). One explanation for the relative dominance of significant HbO2-EEG couplings at electrode position C3 on the left hemisphere could be that all subjects were right-handed. Such right-handers display a clear preponderance of the dynamics of sensorimotor rhythms in the left hemisphere [44]. Although the variability is large, it is remarkable that similar delays between HbO2 peaks and EEG power maxima (3.6 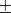 0.9 s [19]) and the delay of the cross correlation maximum from zero (3.7 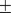 2.5 s) are obtained with 2 completely different methods (see the last paragraph of the Introduction). This gives some support for the robustness of the results.

### Choice of EEG Bands and Interpretation of Short-lasting Increases of Central Alpha and/or Beta Power

The classical central mu rhythm ERD is present in the 9–13 Hz band but mu event-related synchronisation (ERS) during inhibitory control of acquired motor programs is often found between 12–14 Hz [45]. Therefore, we studied the extended alpha band, 9–14 Hz. The central post-movement beta ERS (beta rebound), a short-lasting beta burst after termination of movement or somatosensory stimulation ([3], [46]) shows area specific resonance frequencies: lower beta components (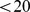 Hz) are predominantly found over the hand area (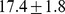 Hz), whereas upper beta components (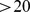 Hz) show the largest frequency components at mid-central sites overlaying the foot representation area and supplementary motor area (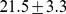 Hz; [47]). To have only one beta band we selected the 15–25 Hz range for our study.

An interesting finding is the temporary periodic increase of alpha and/or beta power during rest. How can this be interpreted? Various studies with combinations of EEG and trans-cranial magnetic stimulation (TMS) [45] and fMRI and EEG ([35], [48]) have shown that a short-lasting increase of central alpha (mu) activity can be related to deactivation or inhibition in sensorimotor structures. From TMS studies ([49] and [50]) we have further evidence that the excitability of corticospinal structures is reduced during the generation of the beta rebound. Summarising these observations, we can speculate that the temporary periodic increase of central alpha and/or beta power in the resting brain can be viewed as the result of a periodic decrease of the excitability level in sensorimotor areas. The opposite, an alpha and/or beta power decrease (desynchronisation), can be viewed as an electrophysiological correlate of an activated cortical network involved in information processing [51]. Therefore, we hypothesise that the observed slow fluctuations of central EEG band power following the prefrontal HbO2 oscillations in the resting brain represent slow intrinsic fluctuations of the excitability level in sensorimotor areas. Recently, it has been shown that very slow EEG fluctuations (0.01–0.1 Hz) are related to cognitive processes and can predict the dynamics of stimulus detection in humans [10].

### Conclusion

Intrinsic prefrontal HbO2 oscillations with frequencies of approximately 0.1 Hz in the resting brain can be coupled, for short time periods of approximately 100 s, with EEG alpha and/or beta power oscillations in sensorimotor areas. We therefore suggest that a coupling between HbO2/Hb and EEG oscillations is an important feature of the resting brain not only found in infants [14] but also in adults. It has to be explicitly mentioned, that a prerequisite for the study was a reliable phase coupling (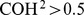) between HbO2 and Hb, whereby such a coupling was found only in a relative small portion of subjects. In the case of a high phase coupling between BP and HbO2 oscillations (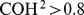) it can be speculated that the slow BP oscillations (Mayer waves) are the driving force for temporary central EEG band power oscillations. The temporary phase coupling between HbO2 and EEG band power oscillations provides further evidence that resting state networks can fluctuate with frequencies between 0.01 and 0.1 Hz [17].

## References

[1] Haggard P (2005) Conscious intention and motor cognition. Trends in cognitive sciences 9: 290–5. 15925808 10.1016/j.tics.2005.04.012

[2] Deecke L, Grozinger B, Kornhuber HH (1976) Voluntary finger movement in man: Cerebral potentials and theory. Biological Cybernetics 23: 99–119. 949512 10.1007/BF00336013

[3] Pfurtscheller G, Lopes da Silva F (1999) Event-related EEG/MEG synchronization and desynchronization: basic principles. Clinical Neurophysiology 110: 1842–1857. 10576479 10.1016/s1388-2457(99)00141-8

[4] Damen E, Brunia C (1987) Changes in Heart Rate and Slow Brain Potentials Related to Motor Preparation and Stimulus Anticipation in a Time Estimation Task. Psychophysiology 24: 700–713. 3438435 10.1111/j.1469-8986.1987.tb00353.x

[5] Florian G, Stancák A, Pfurtscheller G (1998) Cardiac response induced by voluntary self-paced finger movement. International journal of psychophysiology 28: 273–83. 9545662 10.1016/s0167-8760(97)00075-5

[6] Papakostopoulos D, Banerji N, Pocock P (1990) Performance, EMG, brain electrical potentials and heart rate change during a self-paced skilled motor task in Parkinson’s disease. J Psychophysiol 4: 163.

[7] Darwin C, Ekman P (1999) The Expression of the Emotions in Man and Animals. Harper Collins, London.

[8] Soon CS, Brass M, Heinze HJ, Haynes JD (2008) Unconscious determinants of free decisions in the human brain. Nature neuroscience 11: 543–5. 18408715 10.1038/nn.2112

[9] Lambertz M, Langhorst P (1998) Simultaneous changes of rhythmic organization in brainstem neurons, respiration, cardiovascular system and EEG between 0.05 Hz and 0.5 Hz. Journal of the Autonomic Nervous System 68: 58–77. 9531446 10.1016/s0165-1838(97)00126-4

[10] Monto S, Palva S, Voipio J, Palva JM (2008) Very slow EEG uctuations predict the dynamics of stimulus detection and oscillation amplitudes in humans. The Journal of neuroscience 28: 8268–72. 18701689 10.1523/JNEUROSCI.1910-08.2008 PMC6670577

[11] Zhang R, Zuckerman JH, Giller CA, Levine BD (1998) Transfer function analysis of dynamic cerebral autoregulation in humans. Am J Physiol Heart Circ Physiol 274: H233–241. 10.1152/ajpheart.1998.274.1.h233 9458872

[12] Vanhatalo S, Palva JM, Holmes MD, Miller JW, Voipio J, et al (2004) Infraslow oscillations modulate excitability and interictal epileptic activity in the human cortex during sleep. PNAS 101: 5053–7. 15044698 10.1073/pnas.0305375101 PMC387372

[13] Witte H, Putsche P, Schwab K, Eiselt M, Helbig M, et al (2004) On the spatio-temporal organisation of quadratic phase-couplings in ‘tracé alternant’ EEG pattern in full-term newborns. Clinical Neurophysiology 115: 2308–15. 15351372 10.1016/j.clinph.2004.05.014

[14] Roche-Labarbe N, Wallois F, Ponchel E, Kongolo G, Grebe R (2007) Coupled oxygenation oscillation measured by NIRS and intermittent cerebral activation on EEG in premature infants. NeuroImage 36: 718–727. 17482837 10.1016/j.neuroimage.2007.04.002

[15] Zheng F, Sassaroli A, Fantini S (2010) Phasor representation of oxy- and deoxyhemoglobin concentrations: what is the meaning of out-of-phase oscillations as measured by near-infrared spectroscopy? Journal of biomedical optics 15: 040512. 20799778 10.1117/1.3483466 PMC2941517

[16] Sasai S, Homae F, Watanabe H, Taga G (2011) Frequency-specific functional connectivity in the brain during resting state revealed by NIRS. NeuroImage 56: 252–7. 21211570 10.1016/j.neuroimage.2010.12.075

[17] Mantini D, Perrucci MG, Del Gratta C, Romani GL, Corbetta M (2007) Electrophysiological signatures of resting state networks in the human brain. Proceedings of the National Academy of Sciences of the United States of America 104: 13170–5. 17670949 10.1073/pnas.0700668104 PMC1941820

[18] Pfurtscheller G, Klobassa DS, Altstätter C, Bauernfeind G, Neuper C (2011) About the stability of phase shifts between slow oscillations around 0.1 Hz in cardiovascular and cerebral systems. IEEE transactions on biomedical engineering 58: 2064–71. 21693389 10.1109/TBME.2011.2134851

[19] Pfurtscheller G, Bauernfeind G, Neuper C, Lopes da Silva FH (2012) Does conscious intention to perform a motor act depend on slow prefrontal (de)oxyhemoglobin oscillations in the resting brain? Neuroscience letters 508: 89–94. 22206841 10.1016/j.neulet.2011.12.025

[20] De Boer RW, M Karemaker J, Strackee J (1985) Relationships between short-term blood-pressure uctuations and heart-rate variability in resting subjects. I: A spectral analysis approach. Medical & Biological Engineering & Computing 23: 352–358. 4046655 10.1007/BF02441589

[21] Bauernfeind G, Leeb R, Wriessnegger S, Pfurtscheller G (2008) Development, set-up and first results of a one-channel near-infrared spectroscopy system. Biomedizinische Technik 53: 36–43. 18251709 10.1515/BMT.2008.005

[22] Fuster JM (2001) The prefrontal cortex–an update: time is of the essence. Neuron 30: 319–33. 11394996 10.1016/s0896-6273(01)00285-9

[23] De Boer RW, Karemaker JM, Strackee J (1985) Relationships between short-term blood-pressure uctuations and heart-rate variability in resting subjects. II: A simple model. Medical & Biological Engineering & Computing 23: 359–64. 4046656 10.1007/BF02441590

[24] Barnett A, Wolff R (2005) A time-domain test for some types of nonlinearity. IEEE Transactions on Signal Processing 53: 26–33.

[25] Vogel RM, Shallcross AL (1996) The moving blocks bootstrap versus parametric time series models. Water Resources 32: 1875–1882.

[26] Politis D, Romano J (1992) A circular block-resampling procedure for stationary data. In: LePage R, Billard L, editors, Exploring the limits of bootstrap, Wiley.

[27] Pijn JP, Van Neerven J, Noest A, Lopes da Silva FH (1991) Chaos or noise in EEG signals; dependence on state and brain site. Electroencephalography and Clinical Neurophysiology 79: 371–381. 1718710 10.1016/0013-4694(91)90202-f

[28] Seidel H, Herzel H (1998) Bifurcations in a nonlinear model of the baroreceptor-cardiac reex. Physica D: Nonlinear Phenomena 115: 145–160.

[29] Wagner C (1998) Chaos in the cardiovascular system: an update. Cardiovascular Research 40: 257–264. 9893718 10.1016/s0008-6363(98)00251-x

[30] Schomer L, Lopes de Silva F, editors (2011) Niedermeyer’s electroencephalography: Basic principles, clinical applications, and related fields. Lippincott Williams & Wilkins, 6 edition.

[31] Daly I, Nasuto SJ, Warwick K (2011) Brain Computer Interface control via functional connectivity dynamics. Pattern recognition 45: 2123–2136.

[32] Pfurtscheller K, Bauernfeind G, Müller-Putz GR, Urlesberger B, Müller W, et al (2008) Correlation between EEG burst-to-burst intervals and HR acceleration in preterm infants. Neuroscience letters 437: 103–6. 18440144 10.1016/j.neulet.2008.03.079

[33] Steriade M (1999) Coherent oscillations and short-term plasticity in corticothalamic networks. Trends in neurosciences 22: 337–45. 10407416 10.1016/s0166-2236(99)01407-1

[34] Vanhatalo S, Voipio J, Kaila K (2011) Infraslow EEG activity. In: Schomer D, Lopes da Silva F, editors, Niedermeyer’s Electroencephalography: Basic principles, clinical applications and related topics, Lippincott Williams & Wilkins. 6 edition, 741–747.

[35] De Munck JC, I Gonçalves S, C Faes TJ, A Kuijer JP, W Pouwels PJ, et al (2008) A study of the brain’s resting state based on alpha band power, heart rate and fMRI. NeuroImage 42: 112–121. 18539049 10.1016/j.neuroimage.2008.04.244

[36] Thayer JF, Lane RD (2009) Claude Bernard and the heart-brain connection: further elaboration of a model of neurovisceral integration. Neuroscience and biobehavioral reviews 33: 81–8. 18771686 10.1016/j.neubiorev.2008.08.004

[37] Van Roon AM, M Mulder LJ, Althaus M, Mulder G (2004) Introducing a baroreex model for studying cardiovascular effects of mental workload. Psychophysiology 41: 961–981. 15563349 10.1111/j.1469-8986.2004.00251.x

[38] Wolf M, Wolf U, Toronov V, Michalos A, Paunescu L, et al (2002) Different Time Evolution of Oxyhemoglobin and Deoxyhemoglobin Concentration Changes in the Visual and Motor Cortices during Functional Stimulation: A Near-Infrared Spectroscopy Study. NeuroImage 16: 704–712. 12169254 10.1006/nimg.2002.1128

[39] Preiss G, Polosa C (1974) Patterns of sympathetic neuron activity associated with Mayer waves. The American journal of physiology 226: 724–30. 4817426 10.1152/ajplegacy.1974.226.3.724

[40] Montano N (1996) Presence of vasomotor and respiratory rhythms in the discharge of single medullary neurons involved in the regulation of cardiovascular system. Journal of the Autonomic Nervous System 57: 116–122. 8867094 10.1016/0165-1838(95)00113-1

[41] Perlitz V, Lambertz M, Cotuk B, Grebe R, Vandenhouten R, et al (2004) Cardiovascular rhythms in the 0.15-Hz band: common origin of identical phenomena in man and dog in the reticular formation of the brain stem? Pflügers Archiv : European journal of physiology 448: 579–91. 15138824 10.1007/s00424-004-1291-4

[42] Julien C (2006) The enigma of Mayer waves: Facts and models. Cardiovascular Research 70: 12–21. 16360130 10.1016/j.cardiores.2005.11.008

[43] Claus S, Velis D, Lopes da Silva F, Viergever M, Kalitzin S (2012) High frequency spectral components after Secobarbital: The contribution of muscular origin-A study with MEG/EEG. Epilepsy Research (in press). 10.1016/j.eplepsyres.2012.02.002 22476037

[44] Stancak A, Pfurtscheller G (1996) Event-related desynchronisation of central beta-rhythms during brisk and slow self-paced finger movements of dominant and nondominant hand. Cognitive brain research2 4: 171–183. 10.1016/s0926-6410(96)00031-6 8924046

[45] Hummel F (2002) Inhibitory control of acquired motor programmes in the human brain. Brain 125: 404–420. 11844740 10.1093/brain/awf030

[46] Salmelin R, Hari R (1994) Spatiotemporal characteristics of sensorimotor neuromagnetic rhythms related to thumb movement. Neuroscience 60: 537–50. 8072694 10.1016/0306-4522(94)90263-1

[47] Neuper C, Pfurtscheller G (2001) Event-related dynamics of cortical rhythms: frequency-specific features and functional correlates. International journal of psychophysiology 43: 41–58. 11742684 10.1016/s0167-8760(01)00178-7

[48] Hummel F, Saur R, Lasogga S, Plewnia C, Erb M, et al (2004) To act or not to act. Neural correlates of executive control of learned motor behavior. NeuroImage 23: 1391–401. 15589103 10.1016/j.neuroimage.2004.07.070

[49] Chen R, Yaseen Z, Cohen LG, Hallett M (1998) Time course of corticospinal excitability in reaction time and self-paced movements. Annals of neurology 44: 317–25. 9749597 10.1002/ana.410440306

[50] Chen R, Lozano AM, Ashby P (1999) Mechanism of the silent period following transcranial magnetic stimulation. Experimental Brain Research 128: 539–542. 10541749 10.1007/s002210050878

[51] Lopes da Silva F (1991) Neural mechanisms underlying brain waves: from neural membranes to networks. Electroencephalography and clinical neurophysiology 79: 81–93. 1713832 10.1016/0013-4694(91)90044-5

